# Mapping Quantitative Trait Loci for Soybean Seedling Shoot and Root Architecture Traits in an Inter-Specific Genetic Population

**DOI:** 10.3389/fpls.2020.01284

**Published:** 2020-08-19

**Authors:** Silvas J. Prince, Tri D. Vuong, Xiaolei Wu, Yonghe Bai, Fang Lu, Siva P. Kumpatla, Babu Valliyodan, J. Grover Shannon, Henry T. Nguyen

**Affiliations:** ^1^Division of Plant Sciences, University of Missouri, Columbia, MO, United States; ^2^Plant Biology Division, Noble Research Institute, LLC, Ardmore, OK, United States; ^3^BASF Agricultural Solutions, Morrisville, NC, United States; ^4^Nuseed Americas, Woodland, CA, United States; ^5^Amgen Inc., Thousand Oaks, CA, United States; ^6^Corteva Agriscience™, Johnston, IA, United States; ^7^Department of Agriculture and Environmental Sciences, Lincoln University, Jefferson City, MO, United States

**Keywords:** soybean (*Glycine max*), quantitative trait loci (QTL), shoot and root architecture, inter-specific genetic population, molecular markers, single nucleotide polymorphism, KASP assay

## Abstract

Wild soybean species (*Glycine soja* Siebold & Zucc.) comprise a unique resource to widen the genetic base of cultivated soybean [*Glycine max* (L.) Merr.] for various agronomic traits. An inter-specific mapping population derived from a cross of cultivar Williams 82 and PI 483460B, a wild soybean accession, was utilized for genetic characterization of root architecture traits. The objectives of this study were to identify and characterize quantitative trait loci (QTL) for seedling shoot and root architecture traits, as well as to determine additive/epistatic interaction effects of identified QTLs. A total of 16,469 single nucleotide polymorphisms (SNPs) developed for the Illumina beadchip genotyping platform were used to construct a high resolution genetic linkage map. Among the 11 putative QTLs identified, two significant QTLs on chromosome 7 were determined to be associated with total root length (RL) and root surface area (RSA) with favorable alleles from the wild soybean parent. These seedling root traits, RL (BARC_020495_04641 ~ BARC_023101_03769) and RSA (SNP02285 ~ SNP18129_Magellan), could be potential targets for introgression into cultivated soybean background to improve both tap and lateral roots. The RL QTL region harbors four candidate genes with higher expression in root tissues: Phosphofructokinase (Glyma.07g126400), Snf7 protein (Glyma.07g127300), unknown functional gene (Glyma.07g127900), and Leucine Rich-Repeat protein (Glyma.07g127100). The novel alleles inherited from the wild soybean accession could be used as molecular markers to improve root system architecture and productivity in elite soybean lines.

## Highlights

Wild soybeans are useful genetic resources to improve drought avoidance in cultivated soybean by improving shoot and root architectural traits.

## Introduction

Soybean [*Glycine max* (L.) Merr.] is a major oil crop that plays a key role in food and industrial production (Tran and Mochida, 2010). In terms of global production, USA ranks first with 84.2 million metric tons (33% of total global production) of soybean produced followed by Brazil (29%) and Argentina (19%) (www.soystats.com). Cultivated soybean, *Glycine max*, was domesticated from wild soybean (*Glycine soja* Siebold & Zucc.) more than 5,000 years ago in China (Carter et al., 2004) and underwent two rounds of whole genome duplication (Schmutz et al., 2010). Although the breeding process within cultivated soybean has accelerated genetic gain, it has also narrowed the genetic pool (Carter et al., 2004; Hyten et al., 2006). The reduction in genetic diversity among U.S. cultivars poses a threat to future food security due to anticipated pest incidence and disease outbreaks (Hyten et al., 2006).

Wild soybeans represent a significant genetic resource with many rare alleles that are not present in cultivated soybean accessions (Lee et al., 2008; Mammadov et al., 2018). Advancements in next-generation sequencing (NGS) technologies helped researchers to tap into the valuable wild soybean genome for novel haplotypes (Kim et al., 2012; Joshi et al., 2013). Roots are known to be smaller in wild soybean accessions (Liang et al., 2014; Manavalan et al., 2015; Prince et al., 2015). The present study was aimed at identifying wild soybean alleles that could be utilized to widen the genetic base and improve stress resilience in cultivated soybean. Previous studies using 397 diverse soybean accessions with maturity groups, III to IV (Prince et al., 2018) and III to VI (Prince et al., 2019), revealed that constitutive seedling traits, like total root length, root surface area, and lateral root number, are linked to grain yield under water limitation. Field studies in two different soil types that represent most of the U.S. soybean growing target environments also validated the association of root traits to grain yield under stress (Prince et al., 2016; Prince et al., 2019).

Research that uses a crop’s wild relative was first demonstrated in tomato through identification of a major fruit weight QTL of 2.2 in green- and red-fruited wild tomato species, explaining a phenotypic variation of 30% and 47% and increased fruit size (Alpert et al., 1995; Frary et al., 2000). Introgression of yield-enhancing quantitative trait locus (QTL) from *Glycine soja* in cultivated soybean through a marker-assisted backcross breeding approach increased seed yield (Concibido et al., 2003; Li et al., 2008). Similar introductions of wild species haplotypes into cultivated cereal crops were also proven successful for various agronomic traits (Guo et al., 2013; Placido et al., 2013).

Soybean-growing target environments have been severely affected by drought and flooding stresses, and key physiological and biochemical pathways are the basis of adaptation to these stresses (Valliyodan et al., 2014). Deeper root systems enable soybean to acquire water and result in higher yields under drought condition (Hudak and Patterson, 1995). However, biomass allocation to produce robust root systems under non-stress condition was shown to affect final crop productivity (Boyer and Westgate, 2004). In soybean, specific root morphological and anatomical traits have been reported as adaptive mechanisms that enhance plant performance and productivity under drought (Prince et al., 2016; Prince et al., 2017; Prince et al., 2019) and flooding (Valliyodan et al., 2014) stresses. The association of different root traits and their contributions to drought avoidance are well-established in several crops, including rice (Nguyen et al., 1997; Suji et al., 2012), maize (Tuberosa et al., 2011), wheat (Wasson et al., 2012), common bean (Sponchiado et al., 1989), chickpea (Varshney et al., 2011), and soybean (Hufstetler et al., 2007; Prince et al., 2016; Prince et al., 2017; Prince et al., 2019). In comparison to cereal crops, the information on mapped root QTL in legumes is highly limited. Although many rice root QTLs were mapped, there have been few successful cases of their application in marker-assisted breeding programs (Steele et al., 2006; Suji et al., 2012). However, a major QTL controlling root growth angle was cloned, and the gene “*Dro1”* was identified that could be used in the genetic improvement of drought avoidance in rice (Uga et al., 2013). Identifying rare alleles for root growth and development and understanding the regulation of quantitative trait loci (QTL) governing root traits are essential to improving abiotic stress tolerance in soybean.

Previously in soybean breeding programs, selection of root traits under field conditions is hindered by several practical constraints (Pantalone et al., 1996; Myers et al., 2007). The establishment of associations between seedling root traits like total root length, root surface area, lateral root number, and field root response (narrow root angle and fibrous root score) under water limitation is promising (Prince et al., 2016). A gel-based seedling root imaging platform was built to select for these proxy root traits to predict field root response and hasten large-scale germplasm characterization (Prince et al., 2018). Mapping root QTL and identifying markers associated with root traits will facilitate root trait introgression in breeding programs (Coudert et al., 2010). In the last five years, considerable progress has been made toward mapping QTL for soybean root traits in greenhouse (Rong et al., 2011; Prince et al., 2013; Liang et al., 2014; Manavalan et al., 2015; Prince et al., 2015) and field conditions (Abdel-Haleem et al., 2011). Increase in availability of genomic sequences also enabled researchers to link genes associated with different root architectural traits and identify soybean accessions adaptable to water-limited environments (Prince et al., 2017; Prince et al., 2019). In soybean, most of the root QTL mapping studies have utilized genetic diversity within cultivated soybeans. Recently, our group has successfully identified and mapped QTL for root traits using two inter-specific soybean mapping populations (Manavalan et al., 2015; Prince et al., 2015) and could successfully map wild soybean alleles from PI 407162 that improve root traits in cultivated soybean. This was the first effort to map root QTL in an inter-specific mapping population, and further exploration of novel alleles from wild soybeans is needed to improve drought avoidance in soybean.

In this study, we used an inter-specific mapping population developed from a cross between cv. “Williams 82” and “PI 483460B,” a wild soybean accession, to map novel haplotypes for root architectural trait and identify SNPs associated with major root QTLs towards the crop improvement. The objectives of this study were to identify significant genomic regions for seedling shoot and root architecture traits, identify genes underlying major QTLs and identify potential SNPs as molecular markers for root trait breeding.

## Materials and Methods

### Plant Materials

This study used two soybean accessions: cultivar Williams 82 (Bernard and Cremeens, 1988), which is widely used as a reference genome (Schmutz et al., 2010) in various genetic studies, and PI 483460B, a wild soybean accession originating from China with desirable traits for seed composition, such as high protein content (48%) and high linolenic acid (21%) (http://www.ars-grin.gov). Williams 82 has a long and robust root system in comparison to PI 483460B, which has smaller roots. These parental genotypes significantly differ in shoot traits, root architecture, and many other morphological traits. The phenotypic descriptors of the parental lines are presented in **Table 1**. An inter-specific mapping population was developed from a Williams 82 × PI 483460B cross made at the University of Missouri (MU)’s Delta Research Center in Portageville, Missouri. Following the verification of F_1_ true hybridization, F_2_ seed generation was advanced in the soybean nursery in Costa Rica. One hundred eighty-four F_7:8_ recombinant inbred lines (RIL) were generated using the single seed descent method. These RILs were grown in the Bradford Research and Education Center (BREC) at MU in Columbia, Missouri, in the summer of 2012.

**Table 1:** List of soybean shoot and root architecture traits evaluated in the soybean mapping population (Williams 82/PI483460B).

Trait Abbreviation	Description (units)
SL	Shoot length (cm) measured from soil level to cotyledons
PH	Plant height (cm) measured from soil level to shoot tip
TRL	Tap root length (cm)
RL	Root length (cm)
RSA	Root surface area (cm^2^)
RV	Root volume (cm^3^)
RD_L	Distribution of root length in diameter class (1.0–1.5 mm)
RD_S	Distribution of root surface area in diameter class (1.0–1.5 mm)
RD_T	Distribution of root thickness in diameter class (1.0–1.5 mm)

### Phenotyping of RILs

Parental lines (Williams 82 and PI 483460B) and the 184 F_7:8_ RILs were grown in a cone system and replicated five times in a completely randomized block design using DL60L cones and D20 supporting racks (Stuwe and Sons, Oregon, USA). Each replication was conducted separately in the Sears Greenhouse Facility at MU from January 2014 to July 2015 in 20 experimental batches, each comprising 50 genotypes. Turface (Turface Athletics, Illinois, USA) and sand were mixed in a 1:1 ratio as a growing medium.

In the greenhouse, the day and night temperatures were maintained at 29°C and 21°C, respectively. The 12 h photoperiod in the greenhouse was maintained using overhead 400 W metal halide lamps that generated a photosynthetic photon flux density of approximately 1620 μmol m^−2^ s^−1^. The seedlings were grown up to V1 growth stage (approximately 14 days after sowing), and the intact seedlings from the cones were collected to analyze shoot and root traits.

### DNA Extraction

Genomic DNA samples were isolated from pooled leaf tissues of five seedlings of each F_7:8_ RIL and their parents using an automated Autogen 960 system and the CTAB protocol (AutoGen Inc., Holliston, MA) with minor modifications as previously described (Vuong et al., 2010). Briefly, ground leaf tissue was mixed with CTAB extraction buffer, followed by an incubation period at 65°C for 1.5 h. Chloroform was then added to the suspension, followed by agitation and centrifugation. The aqueous layer was collected and treated with RNase enzyme. Following DNA precipitation, DNA pellets were washed with ethanol and dissolved in TE (Tris-HCl-EDTA, pH 8.0) buffer. Subsequently, DNA was quantified and checked for quality.

### SNP Development, Genotyping, and Analysis

A total of 16,469 single nucleotide polymorphism (SNP) markers were included in the final Illumina Infinium BeadChip for genotyping. This SNP set included 7,113 SNPs developed at the Soybean Genomics and Improvement Laboratory at USDA-ARS in Beltsville, Maryland, and 9,356 SNPs developed at the National Center for Soybean Biotechnology (NCSB) in Missouri. DNA samples were quantified by PicoGreen and about 200 to 400 ng DNA/sample were analyzed using the Illumina Infinium assays, following the protocol described by Illumina Inc. (San Diego, CA). SNP calling was automated using the Genome Studio program, with manual modifications when needed. (Infinium^®^ II Assay Workflow, Pub. No. 370-2006-027 07Dec06).

### Construction of Genetic Linkage Map

A genetic linkage map was constructed for the Williams 82 × PI 483460B population (Patil et al., 2018) using the software MSTmap as previously described (Wu et al., 2008). Based on the population size and the number of markers in a genotypic data set, the parameters specified for the MSTmap software were as follows: Kosambi, *p* value cutoff: 1.0E-13 for Genetic mapping function; 2 for No mapping size threshold; 10 cM for No mapping distance threshold; and 0.4 for No mapping missing threshold. The map quality was manually improved by removing markers with significant segregation distortion and misplaced 373 markers compared to the physical map of the Williams 82 reference genome (**Supplementary Table S1**). Out of 9,356 SNPs in the Infimum chips, more than 6,000 markers were found to be polymorphic between the two parents and were incorporated into linkage analysis. The total genetic linkage map distance was 2,925 cM. The number of SNP markers and length of each chromosome are presented in **Supplementary Table S2**.

### Shoot and Root Traits Phenotyping

The mapping population of 184 RILs along with two parental lines of each replication, one normal seedling at the growth stage V1 was collected to manually measure shoot root traits, such as shoot length (SL), plant height (PH), and tap root length (TRL), with a ruler. For other root traits, including total root length (RL), root surface area (RSA), root volume (RV), and distribution of root length (RD_L), root surface area (RD_S), and thickness (RD_T) in diameter class of 1.0 to 1.5 mm, were evaluated using an Epson Scanner 10000XL (Epson America Inc., CA, USA) and analyzed using WinRhizo software (Regent Instruments Inc., Canada). The detailed information of root diameter distribution is critical to understand the root system architecture and its implication in soil function (Blouin et al., 2007). The measurements of shoot- and root-related traits were averaged for further analysis in this study as described in **Table 1**. The phenotypic data generated for the above said traits are provided in **Supplementary Table S3**.

### Gene Expression Data

To gain further insight into the expression of genes underlying root trait QTLs we used the publicly available differential gene expression data of Williams 82, a parental line used in developing this inter-specific mapping population. Gene expressions across diverse soybean genetic backgrounds were obtained from Array express (https://www.ebi.ac.uk/arrayexpress/) and RNA-sequencing datasets (Ithal et al., 2007; Libault et al., 2010; Gong et al., 2014; Kour et al., 2014; Leisner et al., 2014; Lin et al., 2014; Shen et al., 2014; Valdés-López et al., 2014; Wu et al., 2014; Zabala and Vodkin, 2014; Aghamirzaie et al., 2015; Brown and Hudson, 2015; Devi et al., 2015; Huang and Schiefelbein, 2015; Jones et al., 2015; Lambirth et al., 2015; Lanubile et al., 2015; Okamoto et al., 2015; Shin et al., 2015; Whaley et al., 2015; Bellieny-Rabelo et al., 2016; Song et al., 2016; Cho et al., 2017; Dastmalchi et al., 2017; Huang et al., 2017; Waters et al., 2018; Adhikari et al., 2019; Li et al., 2019; Liu et al., 2019; Neupane et al., 2019; Wang et al., 2019) integrated into Genevestigator database, www.genevestigator.com (Zimmermann et al., 2014). The transcriptome data of the wild soybean parental line, PI 483460B used in this study is not available. Thus to mine tissue-specific gene-expression of genes underlying major QTLs, the study used a public transcriptome generated with a *Glycine max*, A81-356022 with introgression of genomic fragments from another wild soybean accession from the same province of China, PI 468916 available in SoyBase (Severin et al., 2010).

### Data Analysis

#### Statistical Analysis

Shoot and root traits among the F_7:8_ RILs and parental lines evaluated were tested for normality using the PROC UNIVARIATE procedure of SAS 9.1 (SAS Institute, Cary, NY, USA) and the Shapiro–Wilk (w) test. Pearson correlation coefficients among the traits were estimated using the SAS PROC CORR procedure. Broad-sense heritability (Nyquist and Baker, 1991) for each trait was estimated based on the expected mean squares (EMS) derived from an analysis of variance (ANOVA) with the SAS PROC GLM procedure. The chromosome numbers (Chr.) were assigned to the soybean genetic linkage groups (LGs) as enumerated in SoyBase (http://www.soybase.org).

#### QTL Mapping

**Figure 1:**
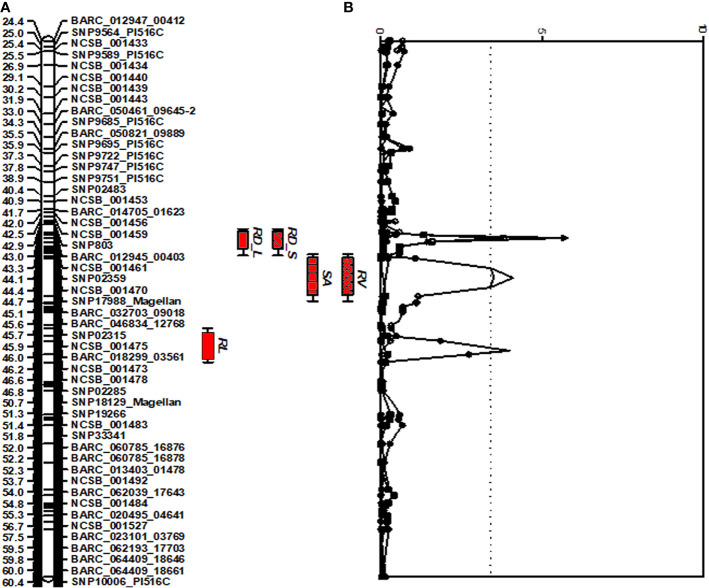
Distribution of QTLs for seedling traits identified in the interspecific soybean mapping population (Williams 82/PI483460B) **(A)** QTLs associated with root traits identified on chromosome 7 with positive allele from wild soybean parent, PI 483460B. **(B)** QTLs with an Likelihood of Odds (LOD) score exceeding the genome-wide LOD of 3.4 were declared as significant QTLs. QTL for root length (RL), root distribution based on length in diameter (1.0–1.5 mm) (RD_L), root distribution based on surface area in diameter (1.0–1.5 mm) (RD_S), root surface area (RSA); and root volume (RV).

A comprehensive approach for QTL analysis, including interval mapping (IM), cofactor selection, genome-wide permutation test, and multi-QTL method (MQM), to detect and map significant QTL was performed using the program MapQTL 5.0 (Van Ooijen and Voorrips, 2001) as previously described (Vuong et al., 2010). A permutation test (Churchill and Doerge, 1994) was performed with 1,000 runs to determine the P = 0.05 genome-wide significance level for declaring a QTL significant. A LOD threshold of 3.4 was determined using a genome-wide permutation test (P = 0.05) for the traits studied and the root trait QTLs detected are shown in **Figure 1**.

A multivariate ANOVA model in SAS (SAS Institute, Cary, NY, USA) was used to estimate the total phenotypic variation explained by the significant QTL. The chromosomes with LOD plots were subsequently created using the MapChart 2.2 program (Voorrips, 2002) based on the outputs from MapQTL 5.0. The prediction of epistatic interactions between significant QTL was performed using the computer program QTLNetwork 2.0 (Yang et al., 2007) with a mixed-model based composite interval mapping (MCIM). For MCIM, critical F-value was assessed by permutation test using 1,000 permutations. QTL effects were estimated using Markov chain Monte Carlo method. Candidate interval selection, epistatic effects, and putative QTL detection were calculated with an experimental-wise type I error of α = 0.05, α = 0.001, and α = 0.001, respectively.

## Results

### Phenotypic Variation of Shoot and Root Architecture Traits

**Figure 2:**
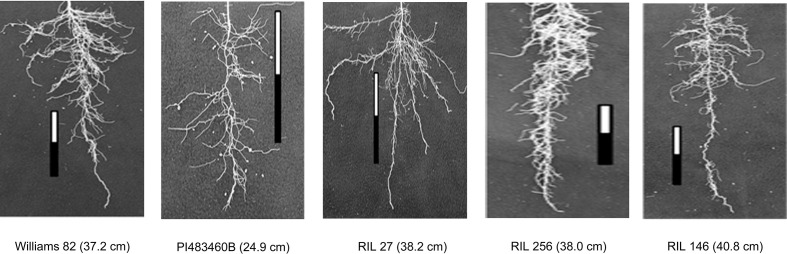
Transgressive segregation pattern for Tap Root Length (TRL) among recombinant inbred lines of the interspecific mapping population. The TRL (in cm) phenotypic values of parental and progenies are provided within the parenthesis.

The parental genotypes, Williams 82 and PI 483460B, showed significant variation (p value= 0.05) for shoot and root traits measured (**Table 2**). Williams 82 is an adapted cultivar with robust root architecture relative to that of the wild soybean parent, PI 483460B. The RIL mapping population developed from a cross of these parental lines showed a transgressive segregation for shoot-related traits (SL, PH) and root traits like TRL (**Table 2**; **Figure 2**). The RILs had longer or shorter shoot and root morphology compared to the *G. max* (Williams 82) and wild parent (PI 483460B), respectively (**Table 2**), for example: TRL (**Figure 2**) and RL. A Shapiro–Wilk test showed that the frequency distributions of the traits followed approximately normal distribution. The heritability of measured traits was calculated based on the analysis of variance of family means. Heritability values ranged from 0.47 to 0.80 for shoot- and root-related traits (**Table 2**). In this mapping population, the significant negative relationship between SL and root traits like TRL and RL showed more allocation of seed reserves to shoot growth and formation of fine roots in contrast to thicker roots, which is evident with its positive association with RD_T (**Table 3**). Even at later stages of shoot development, the trait PH showed a positive relation with RD_T, representing biomass allocation to formation of fine roots. Among root-related traits, TRL is the only trait that showed significant positive correlation with all other root-related traits measured in our study, while RD_T showed positive correlations with TRL and RD_L (**Table 3**). This positive correlation facilitates the selection of longer taproot and robust root system with finer roots, which would enhance both water and nutrients uptake, respectively.

**Table 2:** Descriptive statistics of different shoot and root traits in mapping population derived from Williams 82 X PI 483460B cross.

Trait (†)	Parents		RIL population			Variance	SE*	Skewness	Kurtosis	W-test	*P*-value	Heritability (broad-sense)
	Williams 82	PI 483460B	Mean	Min	Max							
SL	12.48	2.95	7.01	2.80	20.10	9.17	0.11	2.11	9.20	0.8613	<0.0001	0.79
PH	7.28	1.98	4.77	1.68	15.65	10.83	0.12	4.99	42.78	0.6500	<0.0001	0.79
TRL	37.18	27.00	34.08	20.40	41.50	40.37	0.24	−0.72	−0.08	0.9518	<0.0001	0.58
RL	823.73	236.95	451.33	127.61	839.41	30611.63	6.48	0.27	−0.16	0.9914	0.0003	0.80
RSA	130.75	23.52	59.07	16.81	102.56	727.20	1.09	5.19	77.53	0.7730	<0.0001	0.77
RV	1.72	0.20	0.61	0.17	1.19	0.06	0.01	1.00	5.63	0.9537	<0.0001	0.73
RD_L	271.00	55.82	209.20	29.89	382.94	9345.32	3.58	0.18	−0.36	0.9910	0.0002	0.67
RD_S	24.74	5.88	20.14	5.57	34.53	78.21	0.33	0.63	0.89	0.9777	<0.0001	0.64
RD_T	247.75	66.75	148.13	14.25	350.25	10036.33	3.70	1.55	3.30	0.8832	0.000	0.47

(†), trait descriptions were described in **Table 1**, *S.E, Standard Error.

**Table 3:** Pearson correlation coefficients among shoot and root traits evaluated in soybean mapping population.

Trait (†)	SL	PH	TRL	RL	RSA	RV	RD_L	RD_S	RD_T
SL	1	**0.89**	−**0.07**	−**0.11**	−**0.05**	**0.03**	−**0.11**	−**0.11**	**0.06**
		*<.0001*	*0.052*	*0.003*	*0.192*	*0.477*	*0.003*	*0.005*	*0.123*
PH		1	−**0.06**	−**0.12**	−**0.07**	−**0.01**	−**0.12**	−**0.13**	**0.08**
			*0.091*	*0.001*	*0.081*	*0.764*	*0.001*	*0.001*	*0.040*
TRL			1	**0.23**	**0.23**	**0.18**	**0.28**	**0.22**	**0.15**
				*<.0001*	*<.0001*	*<.0001*	*<.0001*	*<.0001*	*<.0001*
RL				1	**0.95**	**0.80**	**0.72**	**0.85**	−**0.08**
					*<.0001*	*<.0001*	*<.0001*	*<.0001*	*0.027*
RSA					1	**0.95**	**0.74**	**0.82**	−**0.05**
						*<.0001*	*<.0001*	*<.0001*	*0.200*
RV						1	**0.68**	**0.71**	**0.02**
							*<.0001*	*<.0001*	*0.558*
RD_L							1	**0.77**	**0.19**
								*<.0001*	*<.0001*
RD_S								1	**0.00**
									*0.938*
									1
RD_T									

(†): trait descriptions were described in **Table 1**. The respective p-value for each trait measured are shown as italicized values.

The + and - bold values represent the positive and negative association between traits.

### QTL for Seedling Shoot and Root Traits

For shoot-related traits, two significant QTLs for SL and PH were detected and mapped to the same regions of chromosomes (Chr.) 3 and 7 with high LOD values of 8.1 and 4.4, explaining a total percent phenotypic variation of 23.6% and 19.8% (**Table 4**). The positive allele for the SL trait on Chr. 3 was provided by the wild soybean parent, PI 438460B, whereas the cultivated soybean, Williams 82, contributed the positive allele for QTL on Chr.7. However, none of the root architectural traits was mapped in the confidence intervals of these shoot-related traits. Two QTLs for TRL were mapped on Chrs. 8 and 20, with LOD values greater than 3, explaining a phenotypic variation of 6.4% and 7.9%, respectively. Similar to the SL QTL, the wild soybean parent contributed the positive allele for PH QTL on Chr. 3. Two SNP markers, NCSB_000550 and SNP5617_Magellan, flanked QTL for RL and RD_L on Chr. 3 and explained more than 7% of phenotypic variation for both traits. Another QTL for RD_L was detected on Chr. 7 (**Figure 1**), which explained a large effect in phenotypic variation of 12.4%, with the positive allele from the wild soybean. The SNP markers, SNP02285 and SNP18129_Magellan, flank the QTL for RSA and RV with the explained phenotypic variation of about 8.9% and 9.9%, respectively, with the positive allele contributed by the wild soybean. Two QTLs for RSA were mapped on Chrs. 3 and 7, with positive allele contributed from both cultivated and wild soybeans, respectively. On Chr. 14, a QTL for RD_T was mapped within a NCSB_003319 ~ SNP21671_PI516C marker interval, explaining a phenotypic variation of 7.3% and with a positive allele from the wild soybean parent. No epistatic interaction was detected between either of the QTL on Chrs. 3 and 7 for SL, PH, TRL, and RV, or other QTL regions mapped on Chrs. 14 and 20. The traits, SL, RL, and RD_L showed inconsistency with their magnitude of additive effects, and phenotypic variation observed might be explained by the nature of gene inheritance and their interactions as explained previously with root (maximum root length) and shoot trait (hypocotyl weight) in soybean seedlings (Liang et al., 2014).

**Table 4:** Quantitative trait loci (QTL) for soybean shoot and root traits were detected with multiple-QTL method in an inter-specific mapping population.

Trait^(a)^	Chr. No^†^	Marker interval	Map position (cM)^‡^	Confidence interval (cM)^§^	LOD score^#^	*R^2^* (%)	Additive effect^††^	Total PVE (%)^‡‡^
SL	3	BARC_038823_07340 ~ NCSB_000710	96.6	95.5_97.2	8.1	17.1	−1.00	23.6
	7	NCSB_001434 ~ NCSB_001439	29.1	26.8_30.2	4.1	8.1	0.68	
PH	3	BARC_038823_07340 ~ NCSB_000710	96.6	95.5_97.2	4.4	9.2	−0.69	19.8
	7	NCSB_001434 ~ NCSB_001439	29.1	26.8_30.2	4.2	9.4	0.70	
TRL	8	BARC_040339_07714 ~ SNP11199_PI516C	50.8	50.5_51.8	3.0	6.4	1.09	16.2
	20	SNP06258 ~ NCSB_004833	82.6	81.8_83.6	3.6	7.9	1.23	
RL	3	NCSB_000550 ~ SNP5617_Magellan	14.4	13.0_15.4	3.4	7.5	35.84	16.2
	7	BARC_020495_04641 ~ BARC_023101_03769	56.7	55.3_57.5	4.0	9.0	−39.60	
RSA	7	SNP02285 ~ SNP18129_Magellan	48.8	46.7_50.6	3.5	8.9	−5.43	
RV	7	SNP02285 ~ SNP18129_Magellan	48.8	46.7_50.6	4.0	9.9	−0.05	
RD_L	3	NCSB_000550 ~ SNP5617_Magellan	14.4	13.0_16.4	3.7	7.9	19.14	18.7
	7	SNP02359 ~ BARC_032703_09018	44.7	44.1_45.1	5.7	12.4	−24.26	
RD_S	3	NCSB_000550 ~ SNP5617_Magellan	14.4	13.0_16.4	4.3	9.5	1.89	16.7
	7	SNP02359 ~ BARC_032703_09018	44.7	44.1_45.1	3.8	8.3	−1.75	
RD_T	14	NCSB_003319 ~ SNP21671_PI516C	138.8	137.6_139.4	3.0	7.3	−17.06	

^†^Chromosome.

^‡^The QTL position was determined based on genetic linkage map constructed in the present study.

^§^Confidence interval for QTL location was estimated using a 2-LOD support interval value as described by van Ooijen (1992).

^#^LOD, logarithm of the odds.

^††^Positive sign indicates alleles from cv. Williams 82 and negative sign indicates alleles from PI 483460B.

^‡‡^PVE, phenotypic variation explained.

^(a)^Trait descriptions were described in **Table 1**.

### Candidate Genes Within Shoot-Related QTL

Generally, wild soybeans are short-statured plants with smaller shoot and root systems as well as smaller leaves and seeds. However, in our study, positive alleles influencing both SL and RL were identified in PI 483460B. Moreover, this wild soybean genotype was found to contribute positive alleles on the Chr. 3 for SL QTL region flanked by BARC_038823_07340 and NCSB_000710 SNP markers, which explained a higher phenotypic variation of 17%. Another QTL for SL was identified on Chr. 7 with the positive alleles from the cultivated soybean, Williams 82 flanked by NCSB_001434 (49,59,463 Mb) - NCSB_001439 (55,22,767 Mb) harbors 65 candidate genes with expression data available for 45 genes in the SoyBase transcriptome database (http://www.soybase.org). Among these genes, four genes expressed higher (more than 50 fold) in leaf tissues with highest expression of protein of unknown function (Glyma.07g060700; 492 fold), followed by 60S ribosomal proteins (Glyma.07g060000; 71 fold and Glyma.07g059900; 61 fold), and core histone protein (Glyma.07g057300; 55 fold).

### Root Trait QTLs With Positive Alleles From Wild Soybean

Four strong effective QTLs were mapped on Chr. 7 for RL, RSA, and RV, RD_L and one QTL for RD_T on Chr. 14. These QTLs were identified with the positive alleles from wild soybean accession. Despite the fact that a small root system is predominant among wild soybeans, the wild soybean accession, PI 483460B, was found to possess positive alleles. These alleles could positively affect total root system architecture, enabling us to effectively mine these QTL regions for candidate genes. These QTLs were flanked by several SNPs: RL (BARC_020495_04641; 14.9 Mb - BARC-023101-03769; 15.3 Mb), both RSA and RV QTL (SNP02285; 0.9 Mb -SNP18129; 10.1 Mb) and RD_L QTL (SNP02359; 88.5 Mb- BARC_032703_09018; 89.3 Mb). The SNPs on Chr. 14 NCSB_003319 (48.2 Mb) - SNP21671_PI516C (48.3 Mb) flank the QTL for RD_T. Based on the major QTL confidence interval flanked by SNPs, genes underlying major QTLs associated with the root phenotypes were identified.

Mining of RL QTL revealed the presence of 27 genes within the confidence interval. Four genes showed higher expression (< 100 fold change) in root tissues: Phosphofructokinase (Glyma.07g126400; 750 fold), Snf7 protein (Glyma.07g127300; 130 fold), unknown functional gene (Glyma.07g127900; 127 fold) and Leucine Rich-Repeat protein (Glyma.07g127100; 219 fold) were identified in the SoyBase transcriptome database (http://www.soybase.org). Mining of whole genome sequence information of parental line PI483460B (Zhou et al., 2015; Patil et al., 2018) within RL QTL interval revealed the wild soybean to have a nonsynonymous SNP in Snf7 protein (Glyma07.G127000) that alters the amino acid from Glutamic acid to Lysine. This gene does not show expression in any of the tissues. However, it has a role in DNA methylation, which is known to alter other gene expression levels (Schmitz et al., 2013). Further, genome mining of the RL QTL region across 106 diverse (landraces and elite) lines sequenced at 17× depth with 10 million SNPs (Valliyodan et al., 2016) revealed two highly expressed genes in root tissues, Phosphofructokinase (Glyma.07g126400) and Leucine Rich-Repeat protein (Glyma.07g127100), harbor non-Synonymous SNP variation that alters amino acid content (**Table 5**). The KASP (competitive allele-specific PCR) assays to genotype these variations are detailed in **Table 5**.

**Table 5:** Details on genes underlying root length (RL) genomic region with non-synonymous SNP variation and KASP assay to genotype sequence level variation.

Gene ID	SNP Position (Mb)	Reference/Alternate allele	Amino acid change	KASP marker assay designed^†^
Glyma.07g126400	15079572	T/A	Aspartic to valine	ATGTCAATTTTAATTGGTTATTTATGAATTGATTTTTAAGTAATTAGGGA**[A/T]** GGTTTGATGGTACTTCTTCATTAAGGTGGTGCTGCATCTCCATTGCTAA
Glyma.07g126400	15079581	A/G	Isoleucine to threonine	TTTAATTGGTTATTTATGAATTGATTTTTAAGTAATTAGGGATGGGTTTG**[A/G]**TGGTACTTCTTCATTAAGGTGGTGCTGCATCTCCATTGCTAAGTATGGAC
Glyma.07g126400	15079825	T/G	Threonine to proline	TCTACCTACCTTAATTTACCCGTCATTTATTTCCATTTTGTTCTGACTTA**[T/G]**GACAGGTCGTTGTTCACAAAGATGGTGCAAGAGGGGTACATTTTAGGCGT
Glyma.07g126400	15079828	G/A	Proline to leucine	CTACCTTAATTTACCCGTCATTTATTTCCATTTTGTTCTGACTTATGACA**[G/A]**GTCGTTGTTCACAAAGATGGTGCAAGAGGGGTACATTTTAGGCGTGCCGG
Glyma.07g127100	15225989	G/C	Isoleucine to methionine	AAGTTGACAATTGACCCATTGTTTTGTTTTCCGACCCTGTTGCCAAGAAA**[G/C]**TATCTTTGGACAATATGCATAGCCATAGCCACCGATATGAAACATCATCA
Glyma.07g127100	15228832	C/T	Glutamic acid to lysine	CACGTTGAGGTATGCCCTGATATAATAGACCTAAAGATGTTTATTTCAAG**[C/T]**CACGTTGAGGTATGCCCTGATATAATAGACCTAAAGATGTTTATTTCAAG
Glyma.07g127100	15230096	G/A	Alanine to valine	AGATGTCTCATGTTTGCAGATGGAAATTCACATTGCTTGAGTTGGAAAAG**[G/A]**AGAATACAGATAGCAATCGATGCTGCAGAGGGTCAGTATAACTTCATTTT
Glyma.07g127100	15230407	A/T	Asparagine to lysine	TACTCAAAAATTTTATGGATTGTTTAAACCAGTCTAAGGGCATGTTTTAT**[A/T]**TTTTTCAACCATGATTATGTTACCATGACTAATAACTTGTTGCCCTGTAA
Glyma.07g127100	15230658	G/T	Leucine to isoleucine	GAAGACTTAGAAGCCAAGATAGCAGATTTTGGCCTCTCCAGGGAGTTTAG**[G/T]**ACAGATAACCAAGATCAACAATCTCAAGTGATTCACAGTGATGCTACAAA

^†^The non-synonymous SNP allele variation between Williams 82 and PI483460B is shown as bold letter within parenthesis and 50-bp sequence on either side of the SNP variation is provided to facilitate the design of KASP marker assay.

**Figure 3:**
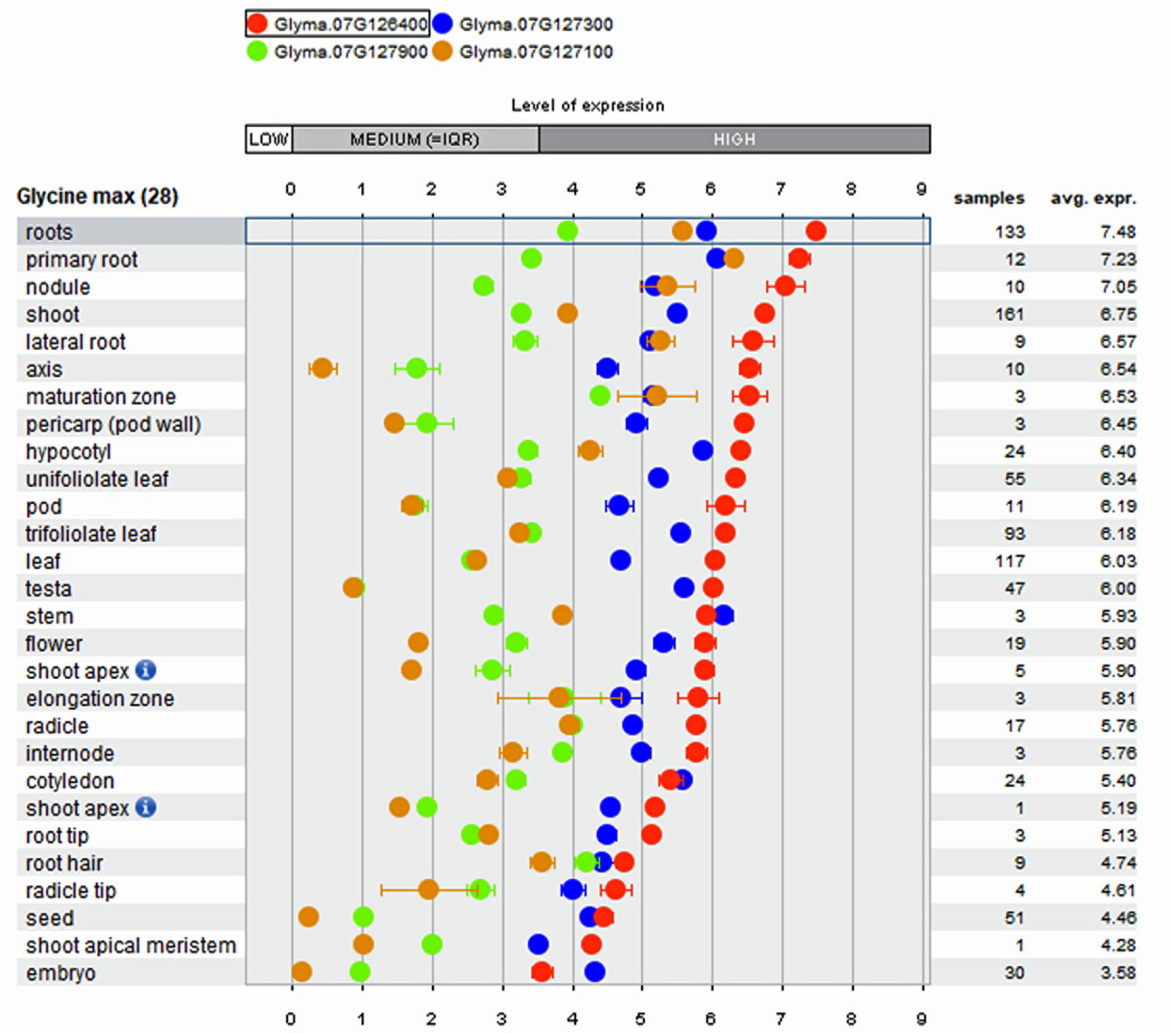
Tissue specific expression pattern of genes within major Root Length (RL) QTL derived from public Affymetrix and RNA-transcriptome experiments integrated into the Genevestigator software.

Array-based transcriptome of diverse soybean genotypes from 31 gene expressions experiments on 28 anatomical tissues revealed that the four genes mentioned above show higher expression in root tissues (**Figure 3**) compared to non-root tissues. The co-location of root QTL for RSA and RV on the same region of Chr. 7 suggests the possibility to improve both traits by identifying key genes underlying the QTL. In comparison to the QTL region on Chr. 7, the QTL confidence interval on Chr. 14 was smaller and observed to possess only 15 genes, of which lysine decarboxylase (Glyma.14g218100; 39 fold) has been reported as expressed specifically higher in root tissues. The biological role of these genes in regulating the roots, main tap, and finer roots warrants further investigation.

## Discussion

### Phenotypic Variation for Shoot and Root Traits

Two parental lines and RILs of the mapping population showed a wide range of phenotypic variation for the shoot and root traits measured along with higher heritability. Cultivar Williams 82 possesses a better developed, robust root system compared to the wild soybean parental line, PI483460B. The recombination of chromosomal regions in the progenies of William 82 and PI 483460B showed higher phenotypic value that exceeded the better parent, William 82 (**Figure 2**). A similar effect was observed in lines of inter-specific mapping populations for root traits, which enabled the identification of novel alleles to improve root system architecture (Manavalan et al., 2015; Prince et al., 2015).

Field-based selection of root traits, root angle, and fibrous root score are proven to contribute to yield protection under stress in soybeans (Fenta et al., 2011; Prince et al., 2016; Prince et al., 2019). The seedlings traits, like total root length, root surface area, lateral root number, were reported to improve root angle and fibrous root score (Prince et al., 2016), which impacted soybean yield under water limitation. In spite of narrow genetic base, intra-specific soybean mapping populations were used to map root QTLs in soybean (Rong et al., 2011; Brensha et al., 2012; Liang et al., 2014). Recent inter-specific mapping studies identified large effect root QTL with positive alleles from wild/semi-wild soybeans (Liang et al., 2014; Manavalan et al., 2015; Prince et al., 2015). Recently, the soybean cultivar USDA‐N7004 was successfully developed with 25% exotic germplasm from the Japanese cultivar Tamahikari (PI 423897) and had improved seed protein and yield on par with check cultivars (McNeece et al., 2020). McCouch et al., 2007 reviewed the use of wild rice, *Oryza rufipogan*, as donors of yield-enhancing alleles to enhance the performance of elite *O. sativa* cultivars.

In this study, we identified nine unique genomic regions significantly associated with shoot and root traits involving two (Chr. 3 and 7) and five chromosomal regions (Chr.3, 7, 8, 14, and 20), respectively. Most of the root-related QTLs with large effects, the positive alleles were contributed by a wild soybean accession, PI 438460B, with a range of phenotypic variation explained from 7.3% to 12.4% for finer root QTL. Interestingly, the TRL QTL on chromosome 8 mapped in this study with a positive allele from Williams 82 collocates with shoot and root weight (fresh and dry) QTLs reported in an intra-specific soybean population (Brensha et al., 2012). This region also collocates with leaf morphology (Kim et al., 2005), internode length (Alcivar et al., 2007), plant height (Lee et al., 1996), and pod number (Zhang et al., 2010) in soybean. The QTL RD_S associated with finer roots collocates with plant height (Guzman et al., 2007), leaf morphology (Orf et al., 1999), and seed yield QTL in soybean (Du et al., 2009).

The root traits of this study showed higher broad-sense heritability, indicating that the major contribution of the phenotypic variation observed was due to largely due to genetic effects. The lines exhibited an especially large phenotypic variation for RL with heritability of 0.8. In soybean, total root length is positively correlated with photosynthetic efficiency at vegetative growth stages and to lateral and fibrous rooting ability as well as canopy temperature during the reproductive growth stage under water-limited conditions in clay soil (Prince et al., 2016). This root trait has been correlated to rooting angle in multiple crop species (Kato et al., 2006; Singh et al., 2011; Kashiwagi et al., 2015) and ultimately cuts down the carbon resources required for the plant to acquire resources in deeper soil horizons in areas with limited rainfall (Wasson et al., 2012).

On chromosome 7, a QTL associated with RSA and RV was identified with a positive allele from wild soybean that explains a higher phenotypic variation. Similar root traits with a positive allele contribution by an inferior parental line in soybean have been reported (Liang et al., 2014; Prince et al., 2015). Rare alleles from wild soybean germplasm (Hajjar and Hodgkin, 2007) have been employed to improve various agronomic traits, like domestication-related traits (Liu et al., 2007), stress tolerance (Carter et al., 2004; Chen et al., 2006; Tuyen et al., 2010), seed compositional traits (Kanamaru et al., 2006), and seed yield (Concibido et al., 2003; Li et al., 2008).

Based on the results obtained, SNPs associated with total root length (RL) on chromosomes 3 (NCSB_000550 ~ SNP5617_Magellan) and 7 (BARC_020495_04641 ~ BARC_023101_03769) and root surface area (RSA) on chromosome 7 (SNP02285 ~ SNP18129_Magellan) are candidates for KASP marker development for use in soybean breeding. Soybean seedling root traits, such as RL and RV are highly associated with stress tolerant (drought and aluminum stress) indices (Liu et al., 2005; Yang et al., 2005; Liu et al., 2007). RL influences the deep rooting ability of soybean, which ultimately improves drought tolerance and is positively associated with yield under drought (Hudak and Patterson, 1995) and rice (Bengough et al., 2011; Suji et al., 2012). Soybean genotypes with deep rooting ability (Taylor et al., 1978; Cortes and Sinclair, 1986) and more fibrous roots (Myers et al., 2007) are supposed to offer effective acquisition of water resources. Root-related traits, such as RL and RSA, facilitate foraging and phosphorus accumulation (Liang et al., 2014) and improve shoot growth (Bates and Lynch, 2001).

### Candidate Genes Underlying QTL

Mining genes that underlie promising QTL with strong effects enable the plant research community to fine-map genes and to identify causal genes responsible for modulating a trait of interest (Pradeepa et al., 2012). Further development in sequencing technologies enables researchers to look for allelic variants among causal genes and to develop functional DNA markers for use in marker-assisted selection (MAS) processes. Similar variants were observed for root traits, such as meristem and cell length in *Arabidopsis* (Meijón et al., 2014), lateral roots in rice (Lyu et al., 2013), soybean (Prince et al., 2019), and root angle in rice (Uga et al., 2013). Most QTL mapping studies in soybean reveal that root traits are polygenic in nature (Liang et al., 2014) and interactions among them (Prince et al., 2015). In this study, we used an inter-specific mapping population in combination with whole-genome transcriptome and Affymetrix gene expression data to identify genes associated with key root traits and to investigate their difference in tissue-specific expression. The Leucine Rich-Repeat class of proteins within RL QTL was found to have large effect on other root traits, like lateral root number and root volume in soybean, on chromosome 7 (Prince et al., 2019). Two SNF proteins identified within this QTL interval with no (Glyma07.G127000) and 130-fold (Glyma.07g127300) expression in root tissues need more detailed analysis to elucidate their roles in root growth and development. In this study, the function of Glyma.07g060700, a gene with unknown function underlying SL QTL on Chr.7, showed a higher level of expression and its interaction with the other genes, Glyma.07g057300, Glyma.07g057200, and Glyma.07g059800 needs to be elucidated to understand the plant stature difference between wild and cultivated soybean species. Further studies on genes underlying the root trait QTLs, RSA, and RL, would enhance the understanding of root growth and development in soybean. The novel wild alleles for these traits could help us to improve root system architecture in cultivated soybean.

## Conclusions

In the present study, we detected and genetically mapped strong effect QTLs for SL and several root-related traits, e.g., root volume and finer root distribution, using an inter-specific mapping population. The QTL region on Chr. 7 governs both RSA and RV with the positive alleles from a wild soybean accession, PI 483460B. These QTL could be potential targets for introgression into cultivated soybean genetic background to improve the root system. Characterizing candidate genes underlying the SL and root traits will enhance understanding of the molecular mechanisms involved in root and shoot growth maintenance. The novel alleles inherited from the wild soybean accession could be potentially used to improve RSA trait in elite, high yielding soybean lines.

## Data Availability Statement

All datasets generated for this study are included in the article/**Supplementary Material**.

## Author Contributions

SP conceived and designed the experiment; phenotypic data generation conducted statistical and QTL mapping analysis; and drafted, wrote, and revised the manuscript. TV performed QTL mapping analysis and edited the manuscript. XW selected a SNP panel and constructed a genetic map. YB, FL, and SK conducted bioinformatics analysis of SNP sequences; designed SNP assays for Infinium beadchips; and performed genotyping analysis. BV, JS, and HN edited the manuscript and provided valuable suggestions.

## Conflict of Interest

Author SP was employed by the company Noble Research Institute, LLC. XW was employed by Bayer Crop Science. YB was employed by Nuseed Americas. FL was employed by Amgen. SK was employed by Corteva Agriscience.

The remaining authors declare that the research was conducted in the absence of any commercial or financial relationships that could be construed as a potential conflict of interest.

## References

[B1] Abdel-Haleem H. Lee G.-J. Boerma R. H. (2011). Identification of QTL for increased fibrous roots in soybean. Theor. Appl. Genet. 122, 935–946. 10.1007/s00122-010-1500-9 21165732

[B2] Adhikari S. Damodaran S. Subramanian S. (2019). Lateral Root and Nodule Transcriptomes of Soybean. Data 4, 64. 10.3390/data4020064

[B3] Aghamirzaie D. Batra D. Heath L. S. Schneider A. Grene R. Collakova E. (2015). Transcriptome-wide functional characterization reveals novel relationships among differentially expressed transcripts in developing soybean embryos. BMC Genomics 16, 928. 10.1186/s12864-015-2108-x 26572793 PMC4647491

[B4] Alcivar A. Jacobson J. Rainho J. Meksem K. Lightfoot D. Kassem M. (2007). Genetic analysis of soybean plant height, hypocotyl and internode lengths. J. Agric. Food Environ. Sci. 1, 1–20.

[B5] Alpert K. Grandillo S. Tanksley S. (1995). fw 2.2: a major QTL controlling fruit weight is common to both red-and green-fruited tomato species. Theor. Appl. Genet. 91, 994–1000. 10.1007/BF00223911 24169988

[B6] Bates T. R. Lynch J. P. (2001). Root hairs confer a competitive advantage under low phosphorus availability. Plant Soil 236, 243–250. 10.1023/A:1012791706800

[B7] Bellieny-Rabelo D. De Oliveira E. Da Silva Ribeiro E. Costa E. P. Oliveira A. E. A. Venancio T. M. (2016). Transcriptome analysis uncovers key regulatory and metabolic aspects of soybean embryonic axes during germination. Sci. Rep. 6, 36009. 10.1038/srep36009 27824062 PMC5099898

[B8] Bengough A. G. Mckenzie B. Hallett P. Valentine T. (2011). Root elongation, water stress, and mechanical impedance: a review of limiting stresses and beneficial root tip traits. J. Exp. Bot. 62, 59–68. 10.1093/jxb/erq350 21118824

[B9] Bernard R. Cremeens C. (1988). Registration of ‘Williams 82’soybean. Crop Sci. 28, 1027–1028. 10.2135/cropsci1988.0011183X002800060049x

[B10] Blouin M. Barot S. Roumet C. (2007). A quick method to determine root biomass distribution in diameter classes. Plant Soil 290, 371–381. 10.1007/s11104-006-9169-1

[B11] Boyer J. Westgate M. (2004). Grain yields with limited water. J. Exp. Bot. 55, 2385–2394. 10.1093/jxb/erh219 15286147

[B12] Brensha W. B. Kantartzi S. K. Meksem K. Grier I. Robert L. Barakat A. (2012). Genetic analysis of root and shoot traits in the ‘Essex’by ‘Forrest’recombinant inbred line (RIL) population of soybean [Glycine max (L.) Merr.]. J. Plant Genome Sci. 1, 1–9. 10.5147/jpgs.2012.0051

[B13] Brown A. V. Hudson K. A. (2015). Developmental profiling of gene expression in soybean trifoliate leaves and cotyledons. BMC Plant Biol. 15, 169. 10.1186/s12870-015-0553-y 26149852 PMC4492100

[B14] Carter T. E. Nelson R. L. Sneller C. H. Cui Z. (2004). “Genetic diversity in soybean. Soybeans: Improvement, production, and uses”, in Agronomy Monographs. 303–416. 10.2134/agronmonogr16.3ed.c8

[B15] Chen Y. Chen P. De Los Reyes B. G. (2006). Differential responses of the cultivated and wild species of soybean to dehydration stress. Crop Sci. 46, 2041–2046. 10.2135/cropsci2005.12.0466

[B16] Cho Y. B. Jones S. I. Vodkin L. O. (2017). Mutations in Argonaute5 illuminate epistatic interactions of the K1 and I loci leading to saddle seed color patterns in Glycine max. Plant Cell 29, 708–725. 10.1105/tpc.17.00162 28351993 PMC5435447

[B17] Churchill G. A. Doerge R. W. (1994). Empirical threshold values for quantitative trait mapping. Genetics 138, 963–971. 7851788 10.1093/genetics/138.3.963 PMC1206241

[B18] Concibido V. La Vallee B. Mclaird P. Pineda N. Meyer J. Hummel L. (2003). Introgression of a quantitative trait locus for yield from Glycine soja into commercial soybean cultivars. Theor. Appl. Genet. 106, 575–582. 10.1007/s00122-002-1071-5 12595984

[B19] Cortes P. Sinclair T. (1986). Water Relations of Field-Grown Soybean under Drought 1. Crop Sci. 26, 993–998. 10.2135/cropsci1986.0011183X002600050031x

[B20] Coudert Y. Périn C. Courtois B. Khong N. G. Gantet P. (2010). Genetic control of root development in rice, the model cereal. Trends Plant Sci. 15, 219–226. 10.1016/j.tplants.2010.01.008 20153971

[B21] Dastmalchi M. Chapman P. Yu J. Austin R. S. Dhaubhadel S. (2017). Transcriptomic evidence for the control of soybean root isoflavonoid content by regulation of overlapping phenylpropanoid pathways. BMC Genomics 18, 70. 10.1186/s12864-016-3463-y 28077078 PMC5225596

[B22] Devi M. J. Sinclair T. R. Taliercio E. (2015). Comparisons of the effects of elevated vapor pressure deficit on gene expression in leaves among two fast-wilting and a slow-wilting soybean. PLoS One 10, e0139134. 10.1371/journal.pone.0139134 26427064 PMC4591296

[B23] Du W. Wang M. Fu S. Yu D. (2009). Mapping QTLs for seed yield and drought susceptibility index in soybean (Glycine max L.) across different environments. J. Genet. Genomics 36, 721–731. 10.1016/S1673-8527(08)60165-4 20129399

[B24] Fenta B. A. Schluter U. Garcia B. M. Duplessis M. Foyer C. H. Kunert K. J. (2011). Identification and application of phenotypic and molecular markers for abiotic stress tolerance in soybean. Soybean—Genetics and Novel Techniques for Yield Enhancement. (nTechOpen), pp. 181–200.

[B25] Frary A. Nesbitt T. C. Frary A. Grandillo S. Van Der Knaap E. Cong B. (2000). fw2. 2: a quantitative trait locus key to the evolution of tomato fruit size. Science 289, 85–88. 10.1126/science.289.5476.85 10884229

[B26] Gong W. Qi P. Du J. Sun X. Wu X. Song C. (2014). Transcriptome analysis of shade-induced inhibition on leaf size in relay intercropped soybean. PLoS One 9, e98465. 10.1371/journal.pone.0098465 24886785 PMC4041726

[B27] Guo S.-B. Yu W. Li X.-Q. Liu K.-Q. Huang F.-K. Chen C.-H. (2013). Development and identification of introgression lines from cross of Oryza sativa and Oryza minuta. Rice Sci. 20, 95–102. 10.1016/S1672-6308(13)60111-0

[B28] Guzman P. S. Diers B. Neece D. St Martin S. Leroy A. Grau C. (2007). QTL associated with yield in three backcross-derived populations of soybean. Crop Sci. 47, 111–122. 10.2135/cropsci2006.01.0003

[B29] Hajjar R. Hodgkin T. (2007). The use of wild relatives in crop improvement: a survey of developments over the last 20 years. Euphytica 156, 1–13. 10.1007/s10681-007-9363-0

[B30] Huang L. Schiefelbein J. (2015). Conserved gene expression programs in developing roots from diverse plants. Plant Cell 27, 2119–2132. 10.1105/tpc.15.00328 26265761 PMC4568505

[B31] Huang L. Shi X. Wang W. Ryu K. H. Schiefelbein J. (2017). Diversification of root hair development genes in vascular plants. Plant Physiol. 174, 1697–1712. 10.1104/pp.17.00374 28487476 PMC5490906

[B32] Hudak C. Patterson R. (1995). Vegetative growth analysis of a drought-resistant soybean plant introduction. Crop Sci. 35, 464–471. 10.2135/cropsci1995.0011183X003500020031x

[B33] Hufstetler E. V. Boerma H. R. Carter T. E. Earl H. J. (2007). Genotypic variation for three physiological traits affecting drought tolerance in soybean. Crop Sci. 47, 25–35. 10.2135/cropsci2006.04.0243

[B34] Hyten D. L. Song Q. Zhu Y. Choi I.-Y. Nelson R. L. Costa J. M. (2006). Impacts of genetic bottlenecks on soybean genome diversity. Proc. Natl. Acad. Sci. 103, 16666–16671. 10.1073/pnas.0604379103 17068128 PMC1624862

[B35] Ithal N. Recknor J. Nettleton D. Maier T. Baum T. J. Mitchum M. G. (2007). Developmental transcript profiling of cyst nematode feeding cells in soybean roots. Mol. Plant-Microbe Interact. 20, 510–525. 10.1094/MPMI-20-5-0510 17506329

[B36] Jones S. I. Tan Y. Shamimuzzaman M. George S. Cunningham B. T. Vodkin L. (2015). Direct detection of transcription factors in cotyledons during seedling development using sensitive silicon-substrate photonic crystal protein arrays. Plant Physiol. 167, 639–649. 10.1104/pp.114.253666 25635113 PMC4348770

[B37] Joshi T. Valliyodan B. Wu J. Lee S. Xu D. Nguyen H. T. (2013). Genomic differences between cultivated soybean, G. max and its wild relative G. soja. BMC Genomics 14, S5. 10.1186/1471-2164-14-S1-S5 PMC3549820 23368680

[B38] Kanamaru K. Wang S. Abe J. Yamada T. Kitamura K. (2006). Identification and characterization of wild soybean (Glycine soja Sieb. et Zecc.) strains with high lutein content. Breed. Sci. 56, 231–234. 10.1270/jsbbs.56.231

[B39] Kashiwagi J. Krishnamurthy L. Purushothaman R. Upadhyaya H. Gaur P. Gowda C. (2015). Scope for improvement of yield under drought through the root traits in chickpea (Cicer arietinum L.). Field Crops Res. 170, 47–54. 10.1016/j.fcr.2014.10.003

[B40] Kato Y. Abe J. Kamoshita A. Yamagishi J. (2006). Genotypic variation in root growth angle in rice (Oryza sativa L.) and its association with deep root development in upland fields with different water regimes. Plant Soil 287, 117–129. 10.1007/s11104-006-9008-4

[B41] Kim H. Kang S. Suh D. (2005). Analysis of quantitative trait loci associated with leaflet types in two recombinant inbred lines of soybean. Plant Breed. 124, 582–589. 10.1111/j.1439-0523.2005.01152.x

[B42] Kim K. S. Diers B. W. Hyten D. L. Roufmian M. A. Shannon J. G. Nelson R. L. (2012). Identification of positive yield QTL alleles from exotic soybean germplasm in two backcross populations. Theor. Appl. Genet. 125, 1353–1369. 22869284 10.1007/s00122-012-1944-1

[B43] Kour A. Boone A. M. Vodkin L. O. (2014). RNA-Seq profiling of a defective seed coat mutation in Glycine max reveals differential expression of proline-rich and other cell wall protein transcripts. PLoS One 9, e96342. 10.1371/journal.pone.0096342 24828743 PMC4020777

[B44] Lambirth K. C. Whaley A. M. Blakley I. C. Schlueter J. A. Bost K. L. Loraine A. E. (2015). A comparison of transgenic and wild type soybean seeds: analysis of transcriptome profiles using RNA-Seq. BMC Biotechnol. 15, 89. 10.1186/s12896-015-0207-z 26427366 PMC4591623

[B45] Lanubile A. Muppirala U. K. Severin A. J. Marocco A. Munkvold G. P. (2015). Transcriptome profiling of soybean (Glycine max) roots challenged with pathogenic and non-pathogenic isolates of Fusarium oxysporum. BMC Genomics 16, 1089. 10.1186/s12864-015-2318-2 26689712 PMC4687377

[B46] Lee S. Bailey M. Mian M. Carter T. Ashley D. Hussey R. (1996). Molecular markers associated with soybean plant height, lodging, and maturity across locations. Crop Sci. 36, 728–735. 10.2135/cropsci1996.0011183X003600030035x

[B47] Lee J.-D. Yu J.-K. Hwang Y.-H. Blake S. So Y.-S. Lee G.-J. (2008). Genetic diversity of wild soybean (Glycine soja Sieb. and Zucc.) accessions from South Korea and other countries. Crop Sci. 48, 606–616. 10.2135/cropsci2007.05.0257

[B48] Leisner C. P. Ming R. Ainsworth E. A. (2014). Distinct transcriptional profiles of ozone stress in soybean (Glycine max) flowers and pods. BMC Plant Biol. 14, 335. 10.1186/s12870-014-0335-y 25430603 PMC4263021

[B49] Li D. Pfeiffer T. Cornelius P. (2008). Soybean QTL for yield and yield components associated with Glycine soja alleles. Crop Sci. 48, 571–581. 10.2135/cropsci2007.06.0361

[B50] Li M. Cao L. Mwimba M. Zhou Y. Li L. Zhou M. (2019). Comprehensive mapping of abiotic stress inputs into the soybean circadian clock. Proc. Natl. Acad. Sci. 116, 23840–23849. 10.1073/pnas.1708508116 31676549 PMC6876155

[B51] Liang H. Yu Y. Yang H. Xu L. Dong W. Du H. (2014). Inheritance and QTL mapping of related root traits in soybean at the seedling stage. Theor. Appl. Genet. 127, 2127–2137. 10.1007/s00122-014-2366-z 25145446

[B52] Libault M. Farmer A. Joshi T. Takahashi K. Langley R. J. Franklin L. D. (2010). An integrated transcriptome atlas of the crop model Glycine max, and its use in comparative analyses in plants. Plant J. 63, 86–99. 10.1111/j.1365-313X.2010.04222.x 20408999

[B53] Lin F. Zhao M. Baumann D. D. Ping J. Sun L. Liu Y. (2014). Molecular response to the pathogen Phytophthora sojae among ten soybean near isogenic lines revealed by comparative transcriptomics. BMC Genomics 15, 18. 10.1186/1471-2164-15-18 24410936 PMC3893405

[B54] Liu Y. Gai J. Lu H. (2005). Identification of rhizosphere abiotic stress tolerance and related root traits in soybean [Glycine max(L.) Merr.]. Zuo wu xue bao 31, 1132–1137. 16231741

[B55] Liu B. Fujita T. Yan Z.-H. Sakamoto S. Xu D. Abe J. (2007). QTL mapping of domestication-related traits in soybean (Glycine max). Ann. Bot. 100, 1027–1038. 10.1093/aob/mcm149 17684023 PMC2759197

[B56] Liu A. Xiao Z. Li M. W. Wong F. L. Yung W. S. Ku Y. S. (2019). Transcriptomic reprogramming in soybean seedlings under salt stress. Plant Cell Environ. 42, 98–114. 10.1111/pce.13186 29508916

[B57] Lyu J. Zhang S. Dong Y. He W. Zhang J. Deng X. (2013). Analysis of elite variety tag SNPs reveals an important allele in upland rice. Nat. Commun. 4, 2138. 10.1038/ncomms3138 23828614 PMC3715847

[B58] Mammadov J. Buyyarapu R. Guttikonda S. K. Parliament K. Abdurakhmonov I. Kumpatla S. P. (2018). Wild relatives of maize, rice, cotton, and soybean: treasure troves for tolerance to biotic and abiotic stresses. Front. Plant Sci. 9:886. 10.3389/fpls.2018.00886 30002665 PMC6032925

[B59] Manavalan L. P. Prince S. J. Musket T. A. Chaky J. Deshmukh R. Vuong T. D. (2015). Identification of novel QTL governing root architectural traits in an interspecific soybean population. PLoS One 10, e0120490. 10.1371/journal.pone.0120490 25756528 PMC4355624

[B60] McCouch S. R. Sweeney M. Li J. Jiang H. Thomson M. Septiningsih E. (2007). Through the genetic bottleneck: O. rufipogon as a source of trait-enhancing alleles for O. sativa. Euphytica 154, 317–339. 10.1007/s10681-006-9210-8

[B61] McNeece B. T. Bagherzadi L. Carter T. E. Jr. Mian M. (2020). Registration of USDA-N7004 soybean germplasm with good yield, elevated seed protein, and 25% exotic pedigree from Tamahikari. J. Plant Regist., 1–6. 10.1002/plr2.20039

[B62] Meijón M. Satbhai S. B. Tsuchimatsu T. Busch W. (2014). Genome-wide association study using cellular traits identifies a new regulator of root development in Arabidopsis. Nat. Genet. 46, 77–81. 10.1038/ng.2824 24212884

[B63] Myers D. B. Kitchen N. R. Sudduth K. A. Sharp R. E. Miles R. J. (2007). Soybean root distribution related to claypan soil properties and apparent soil electrical conductivity. Crop Sci. 47, 1498–1509. 10.2135/cropsci2006.07.0460

[B64] Neupane S. Mathew F. M. Varenhorst A. J. Nepal M. P. (2019). Transcriptome profiling of interaction effects of soybean cyst nematodes and soybean aphids on soybean. Sci. Data 6, 1–8. 10.1038/s41597-019-0140-4 31341170 PMC6656750

[B65] Nguyen H. T. Babu R. C. Blum A. (1997). Breeding for drought resistance in rice: physiology and molecular genetics considerations. Crop Sci. 37, 1426–1434. 10.2135/cropsci1997.0011183X003700050002x

[B66] Nyquist W. E. Baker R. (1991). Estimation of heritability and prediction of selection response in plant populations. Crit. Rev. Plant Sci. 10, 235–322. 10.1080/07352689109382313

[B67] Okamoto S. Suzuki T. Kawaguchi M. Higashiyama T. Matsubayashi Y. (2015). A comprehensive strategy for identifying long-distance mobile peptides in xylem sap. Plant J. 84, 611–620. 10.1111/tpj.13015 26333921

[B68] Orf J. Chase K. Jarvik T. Mansur L. Cregan P. Adler F. (1999). Genetics of soybean agronomic traits: I. Comparison of three related recombinant inbred populations. Crop Sci. 39, 1642–1651. 10.2135/cropsci1999.3961642x

[B69] Pantalone V. Rebetzke G. Burton J. Carter T. (1996). Phenotypic evaluation of root traits in soybean and applicability to plant breeding. Crop Sci. 36, 456–459. 10.2135/cropsci1996.0011183X003600020039x

[B70] Patil G. Vuong T. D. Kale S. Valliyodan B. Deshmukh R. Zhu C. (2018). Dissecting genomic hotspots underlying seed protein, oil, and sucrose content in an interspecific mapping population of soybean using high-density linkage mapping. Plant Biotechnol. J. 16 (11), 1939–1953. 10.1111/pbi.12929 29618164 PMC6181215

[B71] Placido D. F. Campbell M. Jin J. Cui X. Kruger G. R. Baenziger P. S. (2013). Introgression of novel traits from a wild wheat relative improves drought adaptation in wheat (*Triticum aestivum*). Plant Physiol. 161, 1806–1819. 10.1104/pp.113.214262 23426195 PMC3613457

[B72] Pradeepa N. Priya P. S. Prince K. S. J. Kavitha S. Poornima R. Prabhakar M. S. (2012). In Silico analysis of a consensus QTL for drought resistance in rice. Online J. Bioinf. 13, 1–13.

[B73] Prince S. J. Mutava R. N. Pegoraro C. De Oliveira A. C. Nguyen H. T. (2013). Root characters. Genomics and Breeding for Climate-Resilient Crops (Springer), 67–131. 10.1007/978-3-642-37048-9

[B74] Prince S. J. Song L. Qiu D. Dos Santos J. V. M. Chai C. Joshi T. (2015). Genetic variants in root architecture-related genes in a Glycine soja accession, a potential resource to improve cultivated soybean. BMC Genomics 16, 132. 10.1186/s12864-015-1334-6 25765991 PMC4354765

[B75] Prince S. J. Murphy M. Mutava R. N. Zhang Z. Nguyen N. Kim Y. H. (2016). Evaluation of high yielding soybean germplasm under water limitation. J. Integr. Plant Biol. 58, 475–491. 10.1111/jipb.12378 26172438

[B76] Prince S. J. Murphy M. Mutava R. N. Durnell L. A. Valliyodan B. Shannon J. G. (2017). Root xylem plasticity to improve water use and yield in water-stressed soybean. J. Exp. Bot. 68, 2027–2036. 10.1093/jxb/erw472 28064176 PMC5428998

[B77] Prince S. Das K. Tushar N. Murphy M. Valliyodan B. Desouza G. N. (2018). Prediction of Soybean Root Response in the Field Using Nondestructive Seedling Three-Dimensional Root Features. Plant Phenome J. 1, 1–15. 10.2135/tppj2018.04.0003

[B78] Prince S. J. Valliyodan B. Ye H. Yang M. Tai S. Hu W. (2019). Understanding genetic control of root system architecture in soybean: Insights into the genetic basis of lateral root number. Plant Cell Environ. 42, 212–229. 10.1111/pce.13333 29749073

[B79] Rong Z. Hai-Feng C. Xian-Zhi W. Bao-Duo W. Shui-Lian C. Zhang X.-J. (2011). Analysis of QTLs for root traits at seedling stage in soybean. Acta Agronom. Sin. 37, 1151–1158. 10.1016/S1875-2780(11)60032-1

[B80] Schmitz R. J. He Y. Valdés-López O. Khan S. M. Joshi T. Urich M. A. (2013). Epigenome-wide inheritance of cytosine methylation variants in a recombinant inbred population. Genome Res. 23, 1663–1674. 10.1101/gr.152538.112 23739894 PMC3787263

[B81] Schmutz J. Cannon S. B. Schlueter J. Ma J. Mitros T. Nelson W. (2010). Genome sequence of the palaeopolyploid soybean. Nature 463, 178. 10.1038/nature08670 20075913

[B82] Severin A. J. Woody J. L. Bolon Y.-T. Joseph B. Diers B. W. Farmer A. D. (2010). RNA-Seq Atlas of Glycine max: a guide to the soybean transcriptome. BMC Plant Biol. 10, 160. 10.1186/1471-2229-10-160 20687943 PMC3017786

[B83] Shen Y. Zhou Z. Wang Z. Li W. Fang C. Wu M. (2014). Global dissection of alternative splicing in paleopolyploid soybean. Plant Cell 26, 996–1008. 10.1105/tpc.114.122739 24681622 PMC4001406

[B84] Shin J. H. Vaughn J. N. Abdel-Haleem H. Chavarro C. Abernathy B. Do Kim K. (2015). Transcriptomic changes due to water deficit define a general soybean response and accession-specific pathways for drought avoidance. BMC Plant Biol. 15, 26. 10.1186/s12870-015-0422-8 25644024 PMC4322458

[B85] Singh V. Van Oosterom E. J. Jordan D. R. Hunt C. H. Hammer G. L. (2011). Genetic variability and control of nodal root angle in sorghum. Crop Sci. 51, 2011–2020. 10.2135/cropsci2011.01.0038

[B86] Song B. An L. Han Y. Gao H. Ren H. Zhao X. (2016). Transcriptome profile of near-isogenic soybean lines for β-conglycinin α-subunit deficiency during seed maturation. PLoS One 11, e0159723. 10.1371/journal.pone.0159723 27532666 PMC4988716

[B87] Sponchiado B. White J. Castillo J. Jones P. (1989). Root growth of four common bean cultivars in relation to drought tolerance in environments with contrasting soil types. Exp. Agric. 25, 249–257. 10.1017/S0014479700016756

[B88] Steele K. A. Price A. H. Shashidhar H. E. Witcombe J. R. (2006). Marker-assisted selection to introgress rice QTLs controlling root traits and aroma into an Indian upland rice variety. Theor. Appl. Genet. 112, 208–221. 16208503 10.1007/s00122-005-0110-4

[B89] Suji K. Prince K. S. J. Mankhar P. S. Kanagaraj P. Poornima R. Amutha K. (2012). Evaluation of rice (Oryza sativa L.) near iso-genic lines with root QTLs for plant production and root traits in rainfed target populations of environment. Field Crops Res. 137, 89–96. 10.1016/j.fcr.2012.08.006

[B90] Taylor H. Burnett E. Booth G. (1978). Taproot elongation rates of soybeans. Z. fuer Acker und Pflanzenbau. 146 (1), 33–39.

[B91] Tran L.-S. P. Mochida K. (2010). Functional genomics of soybean for improvement of productivity in adverse conditions. Funct. Integr. Genomics 10, 447–462. 10.1007/s10142-010-0178-z 20582712

[B92] Tuberosa R. Salvi S. Giuliani S. Sanguineti M. C. Frascaroli E. Conti S. (2011). Genomics of root architecture and functions in maize. Root genomics. (Springer), 179–204.

[B93] Tuyen D. Lal S. Xu D. (2010). Identification of a major QTL allele from wild soybean (Glycine soja Sieb. & Zucc.) for increasing alkaline salt tolerance in soybean. Theor. Appl. Genet. 121, 229–236. 10.1007/s00122-010-1304-y 20204319

[B94] Uga Y. Sugimoto K. Ogawa S. Rane J. Ishitani M. Hara N. (2013). Control of root system architecture by DEEPER ROOTING 1 increases rice yield under drought conditions. Nat. Genet. 45, 1097–1102. 10.1038/ng.2725 23913002

[B95] Valdés-López O. Khan S. M. Schmitz R. J. Cui S. Qiu J. Joshi T. (2014). Genotypic variation of gene expression during the soybean innate immunity response. Plant Genet. Resour. 12, S27–S30. 10.1017/S1479262114000197

[B96] Valliyodan B. Van Toai T. T. Alves J. D. De Fátima P Goulart P. Lee J. D. Fritschi F. B. (2014). Expression of root-related transcription factors associated with flooding tolerance of soybean (Glycine max). Int. J. Mol. Sci. 15, 17622–17643. 10.3390/ijms151017622 25268626 PMC4227181

[B97] Valliyodan B. Qiu D. Patil G. Zeng P. Huang J. Dai L. (2016). Landscape of genomic diversity and trait discovery in soybean. Sci. Rep. 6, 23598. 10.1038/srep23598 27029319 PMC4814817

[B98] Van Ooijen J. W. Voorrips R. (2001). JoinMap® 3.0, Software for the calculation of genetic linkage maps (The Netherlands: Plant Research International, Wageningen), 1–51.

[B99] Varshney R. K. Pazhamala L. Kashiwagi J. Gaur P. M. Krishnamurthy L. Hoisington D. (2011). “Genomics and physiological approaches for root trait breeding to improve drought tolerance in chickpea (*Cicer arietinum* L.),” in Root genomics. (Springer), 233–250.

[B100] Voorrips R. (2002). MapChart: software for the graphical presentation of linkage maps and QTLs. J. Heredity 93, 77–78. 10.1093/jhered/93.1.77 12011185

[B101] Vuong T. D. Sleper D. A. Shannon J. G. Nguyen H. T. (2010). Novel quantitative trait loci for broad-based resistance to soybean cyst nematode (Heterodera glycines Ichinohe) in soybean PI 567516C. Theor. Appl. Genet. 121, 1253–1266. 10.1007/s00122-010-1385-7 20559815

[B102] Wang J. Hossain M. S. Lyu Z. Schmutz J. Stacey G. Xu D. (2019). SoyCSN: Soybean context-specific network analysis and prediction based on tissue-specific transcriptome data. Plant Direct 3, e00167. 10.1002/pld3.167 31549018 PMC6747016

[B103] Wasson A. P. Richards R. Chatrath R. Misra S. Prasad S. S. Rebetzke G. (2012). Traits and selection strategies to improve root systems and water uptake in water-limited wheat crops. J. Exp. Bot. 63, 3485–3498. 10.1093/jxb/ers111 22553286

[B104] Waters B. M. Amundsen K. Graef G. (2018). Gene expression profiling of iron deficiency chlorosis sensitive and tolerant soybean indicates key roles for phenylpropanoids under alkalinity stress. Front. Plant Sci. 9:10. 10.3389/fpls.2018.00010 29403520 PMC5780454

[B105] Whaley A. Sheridan J. Safari S. Burton A. Burkey K. Schlueter J. (2015). RNA-seq analysis reveals genetic response and tolerance mechanisms to ozone exposure in soybean. BMC Genomics 16, 426. 10.1186/s12864-015-1637-7 26040850 PMC4456062

[B106] Wu Y. Bhat P. R. Close T. J. Lonardi S. (2008). Efficient and accurate construction of genetic linkage maps from the minimum spanning tree of a graph. PLoS Genet. 4, e1000212. 10.1371/journal.pgen.1000212 18846212 PMC2556103

[B107] Wu F. Price B. W. Haider W. Seufferheld G. Nelson R. Hanzawa Y. (2014). Functional and evolutionary characterization of the CONSTANS gene family in short-day photoperiodic flowering in soybean. PLoS One 9, e85754. 10.1371/journal.pone.0085754 24465684 PMC3897488

[B108] Yang S. Chen J. He X. Yu D. Gai J. (2005). Inheritance of drought tolerance and root traits of seedling in soybeans. Soybean Sci. 24, 275–280.

[B109] Yang J. Zhu J. Williams R. W. (2007). Mapping the genetic architecture of complex traits in experimental populations. Bioinformatics 23, 1527–1536. 10.1093/bioinformatics/btm143 17459962

[B110] Zabala G. Vodkin L. O. (2014). Methylation affects transposition and splicing of a large CACTA transposon from a MYB transcription factor regulating anthocyanin synthase genes in soybean seed coats. PLoS One 9, e111959. 10.1371/journal.pone.0111959 25369033 PMC4219821

[B111] Zhang D. Cheng H. Wang H. Zhang H. Liu C. Yu D. (2010). Identification of genomic regions determining flower and pod numbers development in soybean (Glycine max L.). J. Genet. Genomics 37, 545–556. 10.1016/S1673-8527(09)60074-6 20816387

[B112] Zhou Z. Jiang Y. Wang Z. Gou Z. Lyu J. Li W. (2015). Resequencing 302 wild and cultivated accessions identifies genes related to domestication and improvement in soybean. Nat. Biotechnol. 33, 408–414. 10.1038/nbt.3096 25643055

[B113] Zimmermann P. Bleuler S. Laule O. Martin F. Ivanov N. V. Campanoni P. (2014). ExpressionData-A public resource of high quality curated datasets representing gene expression across anatomy, development and experimental conditions. BioData Min. 7, 18. 10.1186/1756-0381-7-18 25228922 PMC4165432

