# Supplementary material for: Mapping Quantitative Trait Loci for Soybean Seedling Shoot and Root Architecture Traits in an Inter-Specific Genetic Population
Source: Front Plant Sci. 2020 Aug 19;11:1284. doi: 10.3389/fpls.2020.01284 (PMC7466435; doi:10.3389/fpls.2020.01284)
Supplement: Supplementary file 2 [file Table_2.docx]

**Mapping quantitative trait loci for soybean seedling shoot and root architecture traits in an inter-specific genetic population**

Silvas J. Prince^1,2^ . Tri D.Vuong^1^ . Xiaolei Wu^3^ . Yonghe Bai^4^ . Fang Lu^5^ . Siva P. Kumpatla^6^ . Babu Valliyodan^1,7^ . J. Grover Shannon^1^ . Henry T. Nguyen^1, §^

Supplementary Table S2. Number of single nucleotide polymorphism markers mapped to each chromosome, corresponding linkage group (LG), and length of chromosome in the Williams 82 × PI 483460B population.

| No | Chromosome | Linkage Group | Number of SNPs | Length of Chromosome (cM) | Average Interval Marker (cM) |
| --- | --- | --- | --- | --- | --- |
| 1 | Gm01 | D1a | 293 | 131.8 | 0.45 |
| 2 | Gm02 | D1b | 312 | 149.3 | 0.48 |
| 3 | Gm03 | N | 267 | 142.5 | 0.53 |
| 4 | Gm04 | C1 | 267 | 123.4 | 0.46 |
| 5 | Gm05 | A1 | 263 | 135.3 | 0.51 |
| 6 | Gm06 | C2 | 354 | 157.2 | 0.44 |
| 7 | Gm07 | M | 230 | 125.8 | 0.55 |
| 8 | Gm08 | A2 | 380 | 223.2 | 0.59 |
| 9 | Gm09 | K | 361 | 134.5 | 0.37 |
| 10 | Gm10 | O | 318 | 183.9 | 0.58 |
| 11 | Gm11 | B1 | 212 | 142.0 | 0.67 |
| 12 | Gm12 | H | 205 | 121.2 | 0.59 |
| 13 | Gm13 | F | 361 | 146.7 | 0.41 |
| 14 | Gm14 | B2 | 272 | 149.3 | 0.55 |
| 15 | Gm15 | E | 414 | 167.2 | 0.40 |
| 16 | Gm16 | J | 278 | 141.1 | 0.51 |
| 17 | Gm17 | D2 | 281 | 146.7 | 0.52 |
| 18 | Gm18 | G | 509 | 143.9 | 0.28 |
| 19 | Gm19 | L | 241 | 129.1 | 0.54 |
| 20 | Gm20 | I | 300 | 131.4 | 0.44 |
| **Total** |  |  | **6,118** | **2,925.6** |  |
